# Targeting Peroxisome Proliferator-Activated Receptor-β/δ (PPARβ/δ) for Cancer Chemoprevention

**DOI:** 10.1007/s40495-015-0026-x

**Published:** 2015-04-01

**Authors:** Jeffrey M. Peters, Pei-Li Yao, Frank J. Gonzalez

**Affiliations:** 1 Department of Veterinary and Biomedical Sciences and The Center of Molecular Toxicology and Carcinogenesis, The Pennsylvania State University, University Park, PA 16802 USA; 2 Laboratory of Metabolism, National Cancer Institute, Bethesda, MD 20892 USA

**Keywords:** Peroxisome proliferator-activated receptor-β/δ, Cancer, Chemoprevention, Inflammation

## Abstract

The role of peroxisome proliferator-activated receptor-β/δ (PPARβ/δ) in cancer remains contentious due in large part to divergent publications indicating opposing effects in different rodent and human cell culture models. During the past 10 years, some facts regarding PPARβ/δ in cancer have become clearer, while others remain uncertain. For example, it is now well accepted that (1) expression of PPARβ/δ is relatively lower in most human tumors as compared to the corresponding non-transformed tissue, (2) PPARβ/δ promotes terminal differentiation, and (3) PPARβ/δ inhibits pro-inflammatory signaling in multiple in vivo models. However, whether PPARβ/δ is suitable to target with natural and/or synthetic agonists or antagonists for cancer chemoprevention is hindered because of the uncertainty in the mechanism of action and role in carcinogenesis. Recent findings that shed new insight into the possibility of targeting this nuclear receptor to improve human health will be discussed.

## Introduction

Shortly after the initial discovery of the nuclear receptor, peroxisome proliferator-activated receptor-α (PPARα) [1], PPARβ/δ was identified [2, 3]. The physiological roles of PPARβ/δ were elusive, and it was not until 1999 that the first report suggesting that PPARβ/δ was involved with cancer was reported [4]. In this study, the authors suggested that PPARβ/δ was activated by cyclooxygenase II (COX-2)-derived metabolites and promoted tumorigenesis in the colon by increasing cell proliferation [4]. However, since this time, numerous studies have revealed related and different hypotheses resulting in contradictory views and considerable uncertainty surrounding PPARβ/δ and cancer (reviewed in [5–8, 9•]).

A number of mechanisms by which ligand activation of PPARβ/δ influence cancer have been postulated using animal and human models, with some gaining stronger weight of evidence than others (reviewed in [5–8, 9•]). The majority of these mechanisms are dependent on the relative expression of the receptor and include molecular changes that modulate cell cycle progression, programmed cell death, cell survival, immunomodulation, differentiation status, and senescence. The focus of this review is on recent advances made in the past 5 years that are beginning to clarify the feasibility and potential for targeting PPARβ/δ for cancer chemoprevention in humans.

## Expression of PPARβ/δ in Non-transformed Tissues and Cancer

**Fig. 1:**
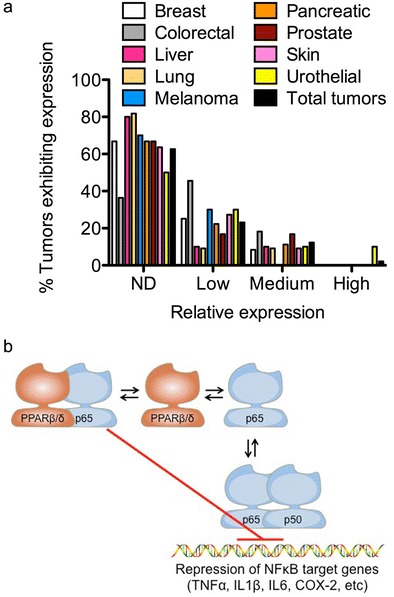
Expression of PPARβ/δ in human tumors and control tissue and mechanism of repression of pro-inflammatory signaling by PPARβ/δ. **a** Relative expression of protein based on analysis from the Human Protein Atlas on June 17, 2014, Version 12, Ensembl version 73.37. Relative expression is depicted as not detected (*ND*), *low*, *medium*, or *high* based on the parameters defined by the Human Protein Atlas. The total number of human tumors examined was 195. The total number of breast tumors examined was 12. The total number of colorectal tumors examined was 11. The total number of liver tumors examined was 10. The total number of lung tumors examined was 11. The total number of melanomas examined was 10. The total number of pancreatic tumors examined was 9. The total number of prostate tumors examined was 12. The total number of skin tumors examined was 11. The total number of urothelial tumors examined was 10. **b** PPARβ/δ can bind with the p65 subunit of NFκB and, by doing so, inhibit the ability of p65 to heterodimerize with the p50 subunit of NFκB, thereby inhibiting expression of NFκB target genes including TNF-α, IL-1β, IL-6, COX-2, etc. This causes inhibition of pro-inflammatory signaling

Quantitative expression patterns of PPARβ/δ have only recently been more precisely determined. For many years, relative expression of PPARβ/δ in human tissues remained obscure due in large part to the lack of highly quantitative approaches and the reliance on less quantitative methodology including simple assessments based primarily on messenger RNA (mRNA) expression (reviewed in [5–8, 9•]). Two publically available databases have been making large advances in elucidating the relative expression of PPARβ/δ in control non-transformed tissues and a variety of cancers. The Human Protein Atlas (www.proteinatlas.org) and Oncomine (www.oncomine.org) represent excellent resources for comparing the relative expression of both mRNA and protein [10••] or mRNA from microarray databases (Oncomine), respectively. Examination of these databases reveals that the expression of PPARβ/δ is relatively high in glandular cells found in the epithelial lining of the small intestine and the colon, glandular cells that compose the ductal cells of the breast, and respiratory epithelial cells in the nasopharynx in the lung, among other cell types in other tissues that also exhibit relatively high expression [10••]. By contrast, in tumor samples examined in The Human Protein Atlas to date (*N* = 192), the relative expression of PPARβ/δ is high to medium in only 2.1 or 12.3 % of all tumors examined, respectively, whereas the relative expression of PPARβ/δ is low to undetectable in 23.1 or 62.6 % of all tumors examined, respectively [10••]. While there is some variation in the relative expression between different tumor types (Fig. 1a), the majority of tumors examined in this database exhibited PPARβ/δ expression in only a fraction of the cells, while malignant cells were in general negative for the expression of PPARβ/δ [10••]. However, it is worth noting that testicular cancers, malignant gliomas, and lymphomas did exhibit relatively strong nuclear staining [10••]. In some cases, the findings observed in The Human Protein Atlas have also been confirmed using highly quantitative approaches including intestinal cancers where markedly reduced expression of PPARβ/δ is found in both human and mouse models [11, 12]. Furthermore, many proteins that can be regulated by PPARβ/δ are also found expressed at negligible to modestly low levels in cancers as compared to non-transformed tissues, including fatty acid-binding protein 1 (FABP1), FABP2, FABP3, FABP4, adipocyte differentiation-related protein, carnitine palmitoyltransferase 1A (CPT1A), CPT1C, and angiopoietin-like 4 [10••]. Results observed at the protein level have also been confirmed at the mRNA level in some cases. For example, the relative expression of PPARβ/δ is lower in human breast cancer samples (*N* = 12) based on analyses reported in The Human Protein Atlas [10••]. The Oncomine database revealed that expression of *PPARβ*/*δ* mRNA is also markedly reduced in human ductal breast adenocarcinomas as compared to normal tissue in three independent studies (*P* ≤ 0.003) [13, 14, 15•]. Thus, there are some consistencies in the literature indicating that the relative expression of PPARβ/δ is reduced in human cancers.

In addition to the consistencies in the reduced expression of PPARβ/δ of different human tumors compared to normal tissue, several recent studies also support these observations. For example, a study examining human colorectal cancer patients revealed that higher expression of PPARβ/δ in primary colorectal tumors was associated with lower expression of a marker of relative cell proliferation (Ki67), a higher frequency of stage I cases in these patients, a lower frequency of later stage cases, and a lower rate of lymph node metastasis [16••]. Moreover, colorectal cancer patients with relatively low expression of PPARβ/δ were ~4 times more likely to die of colorectal cancer than those with a relatively higher expression of PPARβ/δ in primary tumors [16••]. For colon cancer, due in part to the relatively large number of patients examined (141) and the duration of the follow-up (~15 years), this is the best evidence to date supporting the view that PPARβ/δ has tumor suppressor activity. These findings are also similar to results observed in human colon cancer cell lines when expression of PPARβ/δ is knocked down. Reduced expression of PPARβ/δ in KM12C human colon cancer cells causes decreased differentiation and an increased tumor size of xenografts as compared to control xenografts from KM12C cells that express PPARβ/δ [17]. By contrast, another more limited study suggested that the survival of colorectal cancer patients was negatively correlated with expression of both PPARβ/δ and COX-2 in their tumors. Survival of 17 colorectal cancer patients whose tumor samples were positive for both PPARβ/δ and COX-2 expression, based on immunohistochemical analysis, was lower as compared with colorectal patients with tumors that appeared to express only PPARβ/δ, COX-2, or were not immunoreactive for PPARβ/δ and COX-2 [18]. This suggests that PPARβ/δ could cooperatively promote colorectal cancer via an undetermined mechanism that involved COX-2. However, this study has a number of limitations that prevents drawing firm conclusions, including (1) the total number of patients examined was low (52); (2) the follow-up was limited to less than 2 years; (3) the study relied on immunohistochemistry for estimating PPARβ/δ protein expression and correlating with survival, which has inherent problems and is not feasible (as discussed in previous papers [9•, 19]); (4) there was no comparison of patient survival for those with lower versus higher expression of PPARβ/δ alone; and (5) there was no comparison of survival for patients with different-stage disease whose tumors expressed COX-2 only, since this phenotype with early stage I tumors should survive longer than those exhibiting this phenotype with stages II–IV tumors [20]. Thus, there is actually accumulating evidence that the relatively higher expression of PPARβ/δ, similar to that found in normal colonic epithelial cells [10••], is protective against human colon cancer and that agonists that activate this receptor may prove to be chemopreventive for this disease.

A recent study using microarray analysis suggested that higher expression of PPARβ/δ is negatively associated with survival of breast cancer patients [21]. This negative correlation was independent of estrogen receptor (ER) status (i.e., the same effect was noted with ERα-negative and ERα-positive cancer patients), which is in contrast to previous work suggesting that activation of PPARβ/δ in ERα-positive, but not ERα-negative, human breast cancer cells caused increased cell proliferation [22]. However, this study has limitations that prevent drawing firm conclusions, including (1) the authors provide no indication how they defined “low,” “medium,” or “high” expression of *PPARβ*/*δ* mRNA; (2) the study relied on microarray mRNA expression data of PPARβ/δ from a separate study [23] that did not confirm differential mRNA expression and did not examine protein expression in the 295 patients; and (3) the data were not stratified to determine if there were differences in survival that could have been influenced by lymph node-negative disease, lymph node-positive disease, or whether there were differences in survival that were influenced by the use of chemotherapy, hormone therapy, or both chemotherapy and hormone therapy received by 130 of the 295 patients [21]. This study is also at odds with a recent report that examined the effect of over-expressing PPARβ/δ in ERα-negative and ERα-positive human breast cancer cells and found marked inhibition of cell growth, and inhibition of tumorigenicity in xenografts derived from either ERα-negative or ERα-positive human breast cancer cells, which was enhanced by ligand activation of PPARβ/δ compared to controls [24•]. Additionally, another recent study [21] is also inconsistent with previous work suggesting that higher expression of PPARβ/δ is negatively associated with breast cancer, because culturing MCF7 human breast cancer cells inhibits, but does not dose-dependently increase, proliferation in response to the ligand activation of PPARβ/δ by GW0742 [25]. Therefore, despite strong evidence that expression of PPARβ/δ is relatively high in glandular cells of human breast tissue, whether increased expression or decreased expression is prognostic for increased survival in humans remains unclear. However, the fact that expression is relatively high in this tissue as observed in the colon, and appears to decrease in human glandular breast tumors [10••] (Fig. 1a), argues against the notion that this protein could promote tumorigenesis. It is also worth noting that in some cells such as keratinocytes, ligand activation of PPARβ/δ can markedly increase its expression by directly increasing its own transcription [26]. Whether this occurs in other tissues and/or cells could also provide clues to the role of this receptor in carcinogenesis.

## PPARβ/δ Promotes Terminal Differentiation

There are numerous reports that PPARβ/δ and ligands that activate PPARβ/δ can promote terminal differentiation. This has been shown in many different models including keratinocytes, intestinal epithelium, osteoblasts, oligodendrocytes, monocytes, and in colon, breast, and neuroblastoma cancer models (reviewed in [5–7, 9•, 27]). The mechanism(s) that mediate increased terminal differentiation by PPARβ/δ and ligands that activate PPARβ/δ include increased expression of gene products required for terminal differentiation and concomitant inhibition of cell proliferation and/or withdrawal from the cell cycle, effects that are not seen in cells lacking expression of PPARβ/δ (reviewed in [5–7, 9•, 27]). That PPARβ/δ promotes terminal differentiation has not been disputed to date. This is of particular interest because differentiation-inducing agents are known to be potentially useful for cancer chemoprevention [28] and/or cancer chemotherapy [29] due in part to their ability to induce cell cycle arrest [30] and/or enhance the effect of anti-cancer drugs [29], respectively.

## The Anti-inflammatory Activities of PPARβ/δ

Similar to the role of PPARβ/δ in promoting terminal differentiation, it is well established that PPARβ/δ and ligand-activated PPARβ/δ can have potent anti-inflammatory activities in many disease models including cancer (reviewed in [8, 9•, 31–35]). This includes PPARβ/δ-dependent reductions in the expression of pro-inflammatory proteins including COX-2, TNF-α, interleukin 1β (IL-1β), and IL-6. It is also interesting to note that many pro-inflammatory mediators including TNF-α, phorbol esters, and others can all induce expression of PPARβ/δ [36, 37], possibly through an AP1 regulatory site in the promoter of the *PPARβ*/*δ* gene [37]. Does the known increase in expression of PPARβ/δ in response to pro-inflammatory signaling molecules mean that PPARβ/δ promotes inflammation? Quite the contrary; the collective evidence generated in the past 10 years indicates otherwise. There are more than 100 studies to date showing that PPARβ/δ and ligands that activate PPARβ/δ can have potent anti-inflammatory activities in numerous rodent and human disease models (reviewed in [8, 9•, 31–35]). Many independent laboratories have reproduced these effects. This suggests that the reason why pro-inflammatory cytokines such as TNF-α, or those released in response to phorbol ester, increase expression of PPARβ/δ is to counteract the effects of the inflammatory response and potentially lead to resolution of the inflammatory signaling. Indeed, the hypothesis that increased expression and/or activation of PPARβ/δ counteracts the effects of the inflammatory response and causes resolution of inflammatory signaling was postulated in 2008 [7]. Evidence supporting this hypothesis is provided by studies demonstrating that over-expression of PPARβ/δ and/or ligand activation of PPARβ/δ in rat macrophages markedly inhibits lipopolysaccharide-induced expression of pro-inflammatory cytokines including TNF-α, IL-1β, and IL-6 as compared to controls [38]. Further, resolution of inflammation has also been observed by simple administration of the PPARβ/δ ligand GW0742 in an ischemia/reperfusion model of tissue injury [39]. In contrast to more than 100 studies using rodent and human models reported by multiple laboratories, a recent paper suggested that ligand activation of PPARβ/δ induces COX-2 expression in mouse colon [40], similar to the reported increase in COX-2 expression observed in human liver cancer cell lines following treatment with a PPARβ/δ ligand [41, 42]. However, other studies found no change in colonic COX-2 expression in mice by ligand activation of PPARβ/δ [43] and no change in expression of COX-2 in human liver cancer cell lines in response to ligand activation of PPARβ/δ [44]. In fact, ligand activation of PPARβ/δ in mouse macrophages or microglial cells, and rat cardiomyocytes, vascular smooth muscle cells, and cardiomyocytes prevents induced expression of COX-2 in these cell types [45–49]. Thus, despite some conflicting evidence, there is an overwhelming amount of studies demonstrating that PPARβ/δ inhibits pro-inflammatory signaling (reviewed in [8, 9•, 31–35]), and thus could serve as a cancer chemopreventive target.

The mechanism by which PPARβ/δ inhibits pro-inflammatory signaling has been primarily attributed to attenuation of nuclear factor kappa beta (NFκB) signaling (Fig. 1b). This mechanism has been reviewed extensively [8, 9•, 31–35]. Interestingly, all three isoforms of PPARs show some common modes of action for inhibiting inflammation. Moreover, there are additional mechanisms by which PPARβ/δ may inhibit pro-inflammatory signaling (reviewed in [8, 9•, 31–35]). For example, it was recently shown that reducing acetylation of the p65 subunit of NFκB in a human keratinocyte cell line via interactions with AMP kinase and SIRT1 can prevent activation of NFκB following treatment with TNF-α, in response to ligand activation of PPARβ/δ [50]. Whether this and other mechanisms described for PPARs can be used as targets for cancer chemoprevention has not been explored sufficiently. This is of interest to point out because there is evidence that blocking TNF-α signaling [51, 52], COX-2 signaling [53], and/or IL-1β [54, 55] may be suitable for cancer chemoprevention.

## Contemporary Controversies

There are many examples of putative mechanisms mediated by PPARβ/δ in cancer models in which different laboratories have reported opposing results (reviewed in [5–8, 9•]). Reproducibility of mechanistic studies is a problem for all areas of research, which has led to discontinuation of the development of numerous drugs and carries a large cost [56••, 57, 58••, 59]. As noted above, in studies on the role of PPARβ/δ in cancer, there are many examples where reproducibility between laboratories remains an ongoing problem. In some cases, scientific error may be the cause of the lack of reproducibility. For example, it was postulated that all-*trans* retinoic acid activated PPARβ/δ and promoted tumorigenesis due to the increased expression of a putative target gene, 3-phosphoinositide-dependent protein kinase-1 (PDPK1) [60]. However, at least two independent laboratories failed to reproduce these findings, despite extensive approaches that included the use of the same cell type (HaCaT keratinocytes), but also various experiments that should have derived comparable data supporting this putative mechanism [61–63]. These disparities remain unclear, and to date, no other laboratories have ever reported that this mechanism, does or does not, function in HaCaT keratinocytes. There are many other examples of mechanisms that have been described for PPARβ/δ but have not been reproduced by other laboratories (reviewed in [5–8, 9•]). Thus, the targeting of PPARβ/δ for cancer chemoprevention has been hampered because it is not entirely clear that an agonist, an antagonist, or both, would be suitable for cancer chemoprevention. This is indeed disappointing given the nature of nuclear receptors and the fact that PPARs are typically a nodal target that could potentially affect multiple signaling pathways. The targeting of a nodal target such as a PPAR has advantages because targeting single proteins for cancer chemoprevention has proven ineffective [64].

The development of compounds that target PPARβ/δ has also been negatively influenced by alleged scientific misconduct [65••]. For example, Han and colleagues published several manuscripts describing the effects of ligand activation of PPARβ/δ in human lung cancer cell lines that have caused great confusion in this field. The first study reported that ligand activation of PPARβ/δ increased the expression of the prostaglandin E_2_ receptor subtype EP4 via phosphatidylinositide 3-kinase (PI3)/protein kinase B (AKT) signaling in human lung cancer cells [66]. A second study reported that ligand activation of PPARβ/δ increased proliferation of human lung cancer cells via downregulation of the tumor suppressor phosphatase and tensin homolog (PTEN) that was also mediated by PI3/AKT signaling [67]. A third paper from this group suggested that ligand activation of PPARβ/δ increased proliferation of human lung cancer cells via interactions with peroxisome proliferator-activated receptor coactivator γ-1α [68]. Subsequently, the publisher retracted one of these published manuscripts in June 2011, along with two other manuscripts focusing on the effects of fibronectin in lung cells [69–71]. In this case, while the retraction notice did not provide an explanation, another peer-reviewed report indicated that these manuscripts were retracted because of alleged scientific fraud [65••]. Additionally, the other two papers published by Han and colleagues reporting effects of ligand activation of PPARβ/δ in human lung cancer cells were also retracted in 2012, and the retraction notices indicated that data presented in the original articles were either manipulated and/or duplicated in other publications [72, 73]. Interestingly, a follow-up manuscript published by another group indicated that ligand activation of PPARβ/δ had no effect on PTEN or AKT expression and did not increase proliferation of two human lung cancer cell lines [74] as initially reported by Han and colleagues [67]. Despite the fact that the three articles describing the effects of ligand activation of PPARβ/δ in human lung cancer cell lines [66–68] were retracted between July 2011 and May 2012, the retracted articles continue to be cited in the literature to support contentions made by others in their own peer-reviewed papers [75–83]. This illustrates the need for investigators in this field to be cognizant of retracted articles and use caution when using references to support their own research publications. This also highlights a problem that exists not only in the field of PPARβ/δ research but others as well.

## Conclusions

The expression of PPARβ/δ is high in many tissues such as colon, breast, and lung epithelium. This is highly inconsistent with the hypothesis that PPARβ/δ promotes tumorigenesis. In contrast, there is strong evidence from multiple laboratories supporting a higher level of reproducibility of studies showing that PPARβ/δ promotes terminal differentiation and inhibits pro-inflammatory signaling. These collective observations argue strongly that PPARβ/δ functions as a tumor suppressor, rather than a tumor promoter. However, considerable research remains before the precise roles of this nuclear receptor in physiology, diseases, and cancer will be elucidated.
